# Spinal deformities: Evidence and patient safety in management

**DOI:** 10.4102/sajp.v77i2.1757

**Published:** 2021-12-20

**Authors:** Tuğba Kuru Çolak, Hans-Rudolf Weiss

**Affiliations:** 1 Department of Physiotherapy and Rehabilitation, Faculty of Health Sciences, Marmara University, Istanbul, Turkey; 2 Schroth Best Practice Academy, Neu-Bamberg, Germany; 3 Koob Scolitech GmbH, Neu-Bamberg, Germany

The conservative management of patients with spinal deformities demands a deep understanding and special skills from the practitioner, be it the physiotherapist, the orthotist, or the medical physician in charge. Therefore, all professionals involved need to update themselves on a regular basis to be able to advise their patients based on actual knowledge with respect to indications, for physiotherapy, brace treatment and surgery.

Many different physical approaches are offered as being the best possible, but many of these are currently not based on evidence. Scoliosis specific exercises (SSE) are defined as physical approaches which are used for the treatment of scoliosis. However, only pattern-specific exercises with a differential approach to different curve patterns like Schroth and the Monticone method are supported by high-level evidence (Kuru et al. 2016; Monticone et al. 2014; Schreiber et al. 2016). We may call these approaches pattern-specific exercises or pattern specific scoliosis rehabilitation (PSSR).

Brace treatment today can also be regarded as being evidence based (Weinstein et al. 2013; Weiss et al. 2021). However, the results of brace treatment differ widely with respect to success rates (Weiss & Turnbull 2020).

Because of the different treatment approaches within the field of physiotherapy and brace treatment and also the variable success rates of such treatments, the actual state of the art needs to be documented and updated on a regular basis. Patient safety in treatment is affected when approaches with inferior quality are proposed. Inferior methods of physiotherapy may be a waste of time and a waste of a patient’s efforts. For brace treatment in the most vulnerable phase of growth, while low-quality bracing may be unable to stop curve progression, high-quality braces may lead to a fast and stable improvement of the curve and deformity (Weiss & Turnbull 2020). What is of prime importance is that at the end of the growth spurt such improvements are no longer possible. Therefore, during the growth spurt it is important to ensure that no time is wasted and that only those measures with the highest possible success rates are applied.

When conservative approaches fail, surgery is proposed. Spinal fusion is still the gold standard of surgery for spinal deformities, but new non-fusion techniques have been developed to avoid ‘functional amputation’. However, the long-term outcome of surgery in general is still not clear (Weiss & Kaspiris 2020).

To shed some light on the actual standards of treatment, on well evaluated guidelines and classifications and on recent scientific investigations, we have compiled this special issue on spinal deformities with the help of well-known experienced practitioners and scientists with a deep understanding of the topic.

This special issue is called ‘Spinal Deformities – Evidence and Patient Safety in Management’ and therefore, within this issue the articles deal with this specific topic.

First, the current guidelines for the treatment of patients with spinal deformities should be validated. To this end, Elçin Dereli’s working group designed a comprehensive questionnaire that was processed and evaluated by over 100 international specialists. It only took two rounds to achieve the required match. Thus, those affected now can review their own decisions with the help of a comprehensively evaluated guideline and can avoid overtreatment and undersupply.

There are a variety of brace management strategies for treating patients with spinal deformities. Unfortunately, these have very different success rates. So, it is time to streamline and standardise brace treatment. The starting point may be the state-of-the-art article on bracing in which the main types of applications are described, and their success rates are documented.

Even if no evidence for the surgical treatment of patients with spinal deformities is found in some Cochrane studies and systematic reviews, the number of operations performed is steadily increasing. Therefore, we also investigated the question of the current evidence base for surgical treatment and, in a critical assessment, looked for reasons for the high increase in the number of operations.

Spinal curvatures can appear quite differently. In scoliosis-specific treatment, classifications are used to recognise curvature patterns and to be able to treat them individually. The two most common pattern classifications for conservative scoliosis treatment were evaluated and tested for reliability. Not only were the X-ray images evaluated, but also the clinical appearance of the examined patients.

Burçin Akçay’s group investigated the perceptual and cognitive asymmetry in the auditory system in patients with adolescent idiopathic scoliosis. In a large number of neurophysiological studies since the 1980s, asymmetrical innervation patterns or asymmetrical functional patterns have been found in patients with scoliosis. The question of whether these findings are the causes or consequences of scoliosis has not yet been answered. This examination, which looks at the auditory system, thus joins the long series of neurophysiological examinations adding a completely new aspect.

Gok Kandasamy and co-workers dealt with university teaching. They introduced and evaluated the use of a Vision-Based Augmented Reality (VBAR) mobile application to teach students the anatomy and accessory movements of the spine. For physiotherapy students, functional anatomy is of paramount importance. The VBAR achieved significantly better learning results than the conventional approach about this complex issue, which may lead to an improvement in the quality of clinical practice.

Nico Tournavitis’ working group adds an interesting aspect to the current state of knowledge in the field of brace application for patients with scoliosis. In various textbooks you can read that the increase in curvature can be stopped with the help of a brace. Recent findings show that with a high-quality orthosis, final straightening of the curvature is also possible. With their investigations into the changes in the wedge shape of vertebral bodies under brace treatment, the authors substantiate the hypothesis that conservative treatment measures not only allow functional but also structural changes to the vertebral bodies.

Finally, a case report on the conservative treatment of a patient with a rare but prognostically unfavourable form of scoliosis was included in our special edition. From the clinical measurements as well as the numerous clinical pictures, one can see that both the specific corrective physiotherapy and the brace application may be able to influence such large and stiff curvatures.

Two further articles by other working groups were invited. Unfortunately, these could not be included in this special edition.

We would like to thank all the working groups for their efforts and their constant support over the past 12 months! We hope that our readers will find this special edition interesting and a real gain in knowledge.

We must thank the reviewers for their always friendly, fair, and helpful comments. We believe we are speaking on behalf of all our authors when we note that all our work has benefited considerably from the suggestions of the reviewers of the *South African Journal of Physiotherapy*!

We would also like to thank the Editor-in-Chief, Prof. Aimee Stewart, for her constant willingness to support us all with words and deeds. The design and development of this special edition in cooperation with the team of the *South African Journal of Physiotherapy*, the cooperation of clinicians, scientists and the publisher in the team was a very pleasant and enriching experience for us!

Tuğba Kuru Çolak

Hans-Rudolf Weiss
